# Amazing Virulence of Germs

**Published:** 1890-10

**Authors:** 


### THE AMAZING VIRULENCE OF GERMS.

The glove poisoning case in St. Paul, which has recently figured conspicuously in the newspapers, finds a counterpart in one reported in *The Medical Press and Circular*, of "a young Jewess from Kieff, who purchased for a ball a pair of long Danish gloves. While in the middle of a dance she suddenly felt a severe pain in her left wrist, which rapidly became inflamed and swollen. Upon reflection, she remembered to have slightly pricked the wrist with a pin while making her toilet. Subsequently, medical examination showed that the young lady was suffering from carbuncle and blood poisoning, contracted from the glove. The medical men in attendance expressed their conviction that the glove had been made from the skin of an animal suffering from anthrax. Within forty-eight hours their unfortunate patient was dead."

### ORGANIC MATTER FROM SPACE.

Chemists report the recent analysis of two remarkable meteorites. One, which fell at Migheni, Russia, contains about 5 per cent. of organic matter in the shape of a yellow substance closely resembling resin, and also has about 2 per cent. of what is apparently a metallic salt of a new element. The other meteorite fell at Carcote, Chili, and contains not only carbon in organic substances soluble in ether, but also an elementary crystalline form of carbon—dull, black and very hard—which is, in fact, a variety of black diamond. The rapidly lengthening list of meteorites of this class raises the interesting question whether the organic matter really indicates the previous existence of some form of life in these celestial visitors, or whether under certain conditions, such as may exist in the earth's interior, carbon and hydrogen may unite, without the presence of any living organism to form organic substances.

